# Laser acupuncture protocol for essential systemic arterial hypertension: randomized clinical trial^1^

**DOI:** 10.1590/1518-8345.1887.2936

**Published:** 2018-07-16

**Authors:** Raphael Dias de Mello Pereira, Neide Aparecida Titonelli Alvim, Claudia Dayube Pereira, Saint Clair dos Santos Gomes

**Affiliations:** 2 PhD, Professor, Escola de Enfermagem, Centro Federal de Educação Tecnológica Celso Suckow da Fonseca, Rio de Janeiro, RJ, Brazil.; 3 PhD, Associate Professor, Escola de Enfermagem Anna Nery, Universidade Federal do Rio de Janeiro, Rio de Janeiro, RJ, Brazil. Scholarship holder at Conselho Nacional de Desenvolvimento Científico e Tecnológico (CNPq), Brazil.; 4 MSc, RN, Instituto Fernandes Figueiras, Fundação Oswaldo Cruz, Rio de Janeiro, RJ, Brazil.; 5 PhD, Researcher, Instituto Fernandes Figueiras, Fundação Oswaldo Cruz, Rio de Janeiro, RJ, Brazil.

**Keywords:** Nursing Care, Hypertension, Health Technology, Acupuncture

## Abstract

**Objectives:**

to evaluate the efficacy of a laser acupuncture protocol developed and applied by nurses in arterial hypertension patients.

**Method:**

randomized, multicenter, triple-blind and two-armed clinical trial. The sample consisted of 102 participants, 51 per arm, both sexes, aged between 30 and 75 years, undergoing drug therapy for a year or more, with difficulty to control blood pressure, maintaining regular measures >140x90 mmHg. Participants underwent six standard or simulated laser-acupuncture sessions, for 24 minutes, within a period of six weeks. Descriptive analyzes expressed as frequencies of occurrences, means and medians were used, and analysis of the association between variables was performed using Student’s t-test and Anova, using Statistica^®^ software, version 12.0. The significance level was set at 5% (alpha=0.05). The comparison between blood pressure measurements was performed using Student’s t-test for paired samples and Anova for repeated measures.

**Results:**

a significant reduction in systolic (p<0.001) and diastolic (p<0.001) blood pressure was observed among participants in the intervention arm, which was not observed in the simulation arm.

**Conclusion:**

the results have demonstrated the efficacy of the protocol. Reduction and control of blood pressure have been demonstrated, indicating the possibility of using this technology for the care of patientes with essential systemic arterial hypertension. Brazilian Registry of Clinical Trials. UTN: U1111-1177-1811. Clinical Trials NCT02530853.

## Introduction

Essential Systemic Arterial Hypertension (SAH) is an important risk factor, precursor and potential factor for the progression of Cardiovascular Diseases (CVD) and their complications^1^ ^-^ ^3^. A multifactorial condition characterized by a sustained increase in arterial blood pressure levels ≥140 and/or 90 mmHg^2^. 

Blood Pressure (BP) control at levels below 140 mmHg for Systolic Blood Pressure (SBP) and 90 mmHg for Diastolic Blood Pressure (DBP) in patients with stages 1 and 2 hypertension, with low and moderate Cardiovascular (CV) risk, as well as those with stage 3 hypertension, has been strongly recommended as a therapeutic goal^2^. For those with stages 1 and 2, however, with a high CV risk, the recommendation differs, and the maintenance of blood pressure levels below 130x80 mmHg is indicated^2^.

The attainment of such results is influenced by several factors such as adherence to medication treatment, availability of the medication in the public health system, health professional/patient relationship, besides those related to the occurrence of adverse or undesirable events, intake of beverages and foods not recommended, smoking and sedentary lifestyle^1^ ^-^ ^3^. 

The treatment has a multidisciplinary nature and is based on drug and non-drug strategies, and the latter are managed and supported by nurses, especially in the area of basic health care^1^ ^-^ ^3^. 

Acupuncture (AP), an ancient technique of the Traditional Chinese Medicine (TCM), is part of the list of non-drug strategies and has been proposed as a complementary therapy for the control of Chronic Noncommunicable Diseases (CNCD) by the World Health Organization (WHO)^4^ ^-^ ^5^.

Several authors have pointed out AP as a complementary therapy in the control of SAH. The results indicate the possibility of its use for an effective control of blood pressure levels, which can be associated to different modalities of drug therapy and performed in several health care settings^6^ ^-^ ^7^. 

AP is a therapeutic modality of TCM that, through the stimulation of specific points in the body, called acupoints, promotes organic self-regulation to fight several health conditions/diseases^8^.

In SAH, this mechanism of self-regulation is related to the balance of the activity of the renin-angiotensin-aldosterone system, in addition to involving plasma alterations of catecholamines and neurotransmitters, such as noradrenaline, serotonin and endorphin^9^.

This technique can be applied through a non-invasive method, without the use of needles (laser-acupuncture), through a minimally invasive method, with the use of extremely thin needles (traditional acupuncture), or through the association of needle use with electrical stimulation (electroacupuncture ), with these two modalities most often found in clinical studies for the demonstration of its safety and efficacy^5^ ^-^ ^6^.

Laser-Acupuncture (LA) method also shows good safety and efficacy, however, studies on this modality are still incipient, as well as those involving the use of this technology in nursing care^5^ ^,^ ^9^ ^-^ ^10^.

The objective of this study was to evaluate the efficacy of a LA protocol, developed and applied by nurses in patients with SAH. This study was based on the hypothesis that the application of AP as a technology for nursing care promotes a significant decrease in blood pressure levels in hypertensive patients undergoing drug treatment and who find difficulties for the effective control of blood pressure.

## Methodology

A randomized, multicenter, controlled, triple-blind and two-armed clinical trial with a random assignment rate set at 50% per arm. This study was performed at the Laboratory of the Integrated Research-Assistance Programme (Pipa), Anna Nery School of Nursing, Federal University of Rio de Janeiro (EEAN/UFRJ), the coordinating center of the study; and at the family health units linked to the Municipal Health Secretariats of the city of Maricá, in the State of Rio de Janeiro, and the city of Vitória, in the State of Espírito Santo. This study was funded by the Ministry of Science, Technology and Innovation/National Council for Scientific and Technological Development/Ministry of Health (MCTI/CNPq/MS), Public Notice number 007/2013, of the Unified Health System (SUS), specific for studies using integrative and complementary health practices, contributing to the displacement of the research team and for the purchase of equipment and inputs for the development of this study.

Sample size was calculated based on the results of the meta-analysis on the subject^6^, which found a difference of 5.72±14.1 in BP values after the application of AP protocols, with a 95% confidence level and 80% power.

The sample consisted of 102 participants with SAH, regardless of hypertension stage, of both sexes, who met the following inclusion criteria: age between 30 and 75 years, undergoing drug treatment for SAH for a year or more, with difficulty to control blood pressure and maintaining the readings performed by health professionals in the clinic above 140x90 mmHg. Patients with physical activity habits or on weight loss diets, patients undergoing treatment with integrative and complementary health practices (acupuncture, Reiki, auriculotherapy, yoga, meditation, among others) were excluded. Patients with upper or lower limb amputations or amputations in areas located at acupuncture points, patients with cardiovascular complications or lesions in target organs due to hypertension or ongoing oncologic diseases, smokers, alcoholics and pregnant women were also excluded.

Nurses and other professionals from the health teams linked to the participating centers performed the recruitment and selection of participants based on the mentioned inclusion and exclusion criteria, and referred the patients cared for by them to the research team. Once the criteria for inclusion in the study were met, participants were referred to a nursing consultation with a nurse who was part of the research team, trained to obtain and register the variable under analysis. This professional provided guidance on the objectives of the study, risks, benefits, probability of participant being included in the intervention or simulation arm, through their expressed agreement. The participant then read and signed the Informed Consent Form (ICF) with the appropriate explanations when necessary. At the end of the consultation, each participant was given a registration number in the survey and was referred to the random assignment carried out by another team member, specifically designated to this purpose. 

The random assignment was performed by block in a ratio of 1:1, using an outsourced service for the random assignment through the internet (http://www.sealedenvelope.com).

The number assigned to the participants was entered into the system that automatically generated their assignment in one of the study arms, without the interference of participants, ensuring equal and independent inclusion chances, that is, both in Arm A - Intervention (acupuncture) and in Arm B - Control (simulation). Only the research assistant responsible for the random assignment was not blinded, being aware of the assignment groups of each participant and being responsible for the confidentiality of the random assignment sequence, as well as the preparation of the equipment for intervention and simulation throughout the study. Blinding of participants, evaluator nurse responsible for the initial and final nursing consultation, and acupuncturist nurse were ensured during the entire course of the research process. For this purpose, an equipment was used that had the same physical and structural characteristics of the equipment used for intervention, except for the absence of the internal device emitting infrared laser. The random assignment groups were revelead to the participants, the evaluator nurse and the acupuncturist nurse only after completion of all research steps (initial assessment, intervention, final assessment).

Nursing consultation and monitoring of blood pressure levels were carried out during the entire period of the study for the operationalization of the nursing care of participants in both arms. The nursing process guided the nursing consultations, with Martha Elizabeth Rogers’s (1970) science of unitary human beings as theoretical basis. At the end of the intervention period, all participants received guidelines aimed at improving adherence to drug treatment, non-drug treatments (meditation, yoga, acupuncture) and changes in lifestyle.

**Figure 1:**
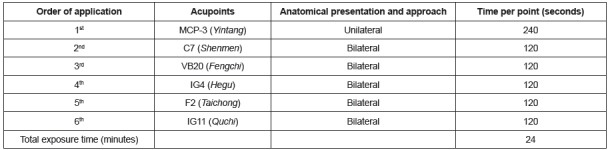
Acupoints used, order of application and dwell time on each laser-acupoint. Rio de Janeiro, RJ, Brazil, 2015

The same protocol was adopted for arms A and B, which was developed based on systematic review studies and specific literature on acupuncture, taking into account the inharmonic patterns related to SAH, according to the TCM/AP. All participants, regardless of the assignment arm, underwent six non-needle interventions, using only a gallium-aluminum-arsenide (Ga-Al-As) low-power infrared laser-acupuncture equipment, with a Nogier frequency of 6 MW, for 24 minutes and an interval of one week between the interventions. The selected acupoints, located in the head (frontal and occipital regions), upper (hands and arms) and lower (feet) limbs, received the direct application of the equipment on the skin. The order of application, points, anatomical presentation and approach (unilateral or bilateral) and the dwell time on each acupoint are shown in Figure 1.

The expected outcome was reducing SBP to levels below 140 mmHg and reducing DBP to levels below 90 mmHg after six sessions. For this purpose, BP was measured before and after each intervention, following a universal measurement protocol: sitting position, 30-minute rest time before interventions, using the indirect oscillometric method, with a precision digital monitor registered at the National Health Surveillance Agency (Anvisa), at the National Institute of Metrology, Quality and Technology (Inmetro), and recommended by the *British Hypertension Society* (BHS). The results were entered into a Microsoft Excel spreadsheet, being under the responsability of the research assistant responsible for the random assignment and subsequently exported to the statistical analysis *software*.

Statistica^®^ *software*, version 12.0, was used for data analysis. Descriptive and association analyzes between variables were used, with a significance level set at 5% (p<0.05). The normal distribution of variables was assessed by the Kolmogorov-Smirnov test (K-S); the comparison between the results of the pre and post-exposure blood pressure measurements was performed using the Student’s t-test for paired samples and Anova was used to assess the different measurements of systolic and diastolic pressures at each moment of the intervention.

The Research Ethics Committee (CEP) of the Ana Nery School of Nursing/São Francisco de Assis Hospital, Federal University of Rio de Janeiro approved this study, with the Opinion number 772.508, registered in the Clinical Trials database (clinicaltrials.gov), available under the code NCT02530853, and in the Brazilian Registry of Clinical Trials (ReBEC), under the code UTN: U1111-1177-1811.

## Results

In total, each arm consisted of 51 participants, who were recruited from September to December 2014, at the research-coordinating center and at the health units of the participating center in the city of Vitória, ES. At the participating center in the city of Maricá, RJ, recruitment was carried out from March to May 2015. The six-week follow-up period took place following the recruitment phase, and the study was completed in September 2015, due to the results. In that month, previous analyzes of the data were performed and the results showed a significant difference between the control and intervention arms. A power of around 100% was found for the sample in the comparison between the moments in both arms. Based on these results, it was decided to discontinue the study, not including any other participants.

Women comprised the majority of the sample, representing 66.7% (n=34) of the participants assigned in arm A and 70.6% (n=36) in arm B. Males represented 33.3% (n=17) of those assigned in arm A and 29.4% (n=15) in arm B.

The mean and median ages of participants were 55.4-53.5 years, respectively, for women and 55.5-55.0 years for men.

The mean BP of study participants was 158.8 mmHg (Standard Deviation - SD=17.4) for SBP and 95.8 mmHg (SD=7.7) for DBP, confirming the difficulty in controlling blood pressure in the sample.

The length of drug treatment of participants was classified into three inclusion ranges: from 1 to 4 years, 36.3% (n=37), from 5 to 10 years, 37.2% (n=38) and from>10 years, 26.5% (n=27).

The use of monotherapy for the treatment of blood pressure of participants was not identified, with combination therapy being the predominant treatment method. The most frequent medications used by participants in both groups were thiazide diuretics (61.8%, n=63), angiotensin II receptor blockers (56.9%, n=58) and the angiotensin converting enzyme inhibitors (29.4%, n=30).

The association between angiotensin II receptor blockers and/or angiotensin converting enzyme inhibitors and thiazide diuretics was identified in 72.5% (n=74) of participants.

**Figure 2:**
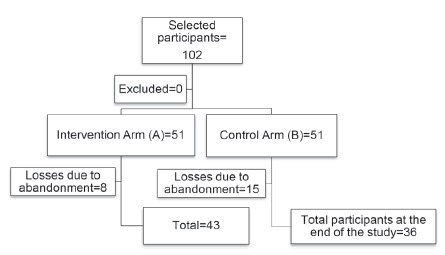
Distribution of participants in the research. Rio de Janeiro, RJ, Brazil, 2015

Throughout the study period, some participants did not attend the visits without removal of their ICF, justifying the participants dropping out due to various reasons such as the need for frequent travel, new job and change of address. This resulted in loss of follow-up in both arms of the study (Figure 2).

In arm A, there was a loss of 15.6% (n=8), while in arm B it was 29.4% (n=15), totaling a loss of 22.3% (n=23).

There was a higher reduction of the sample among women in arm B (simulation), which had a reduction of 33.3% (n=12) when compared to the same gender in arm A (intervention), whose reduction was 14.7 % (n=5).

In the period from August to September 2015, an interim analysis was performed considering 79 participants in total. On that occasion, 23 losses were observed in total, which did not compromise the reliability of the results, as demonstrated by the K-S test for normality, since the null hypothesis (p>0.05) was not rejected in the normal probability distribution.

Although six exposures were scheduled and performed in each arm, considering the non-attendances, the median varied from 5 to 6 exposures, being more regular in arm A, with an average of 5 exposures and SD=1.4±1.3 for the sexes, respectively. These results are summarized in Table 1.

**Table 1:** Distribution profile of participants in the research. Rio de Janeiro, RJ, Brazil, 2015

Arm	Gender	Started exposure			Finished exposure			Reduction (%)		Exposures (mean±SD^†^)	Exposures (median)
		n*	%		n*	%		n*	%		
A	Female	34	100		29	85.3		5	14.7	4.9±1.4	5
	Male	17	100		14	82.4		3	17.6	4.9±1.3	5
B	Female	36	100		24	66.7		12	33.3	4.1±1.9	5
	Male	15	100		12	80.0		3	20.0	4.9±1.7	6
Total		102	100		79	77.5		23	22.5	4.6±1.7	5

*n: Number of participants; †SD: Standard deviation

In the final analysis, it was considered only the total number of participants attending all sessions, that is, 43 participants in arm A and 36 in arm B. It was noted that the study was initiated, in arms A and B, with BP redings above 140x90 mmHg in the pre- and post-intervention moments. In arm A, significant differences were observed in BP levels at different moments of observation, with a trend of constant reduction, reaching a plateau of 128x80 mmHg (at moment 6 in the post-intervention). In arm B, no significant differences were observed in the mean BP values, at the different moments of observation, in the pre- and post-exposure periods, see Table 2.

**Table 2:** Mean SBP and DBP values in pre and post-exposure measurements in both arms of the study. Rio de Janeiro, RJ, Brazil, 2015

Arm	Exposure	Moment	SBP*			DBP^†^	
			Mean±SD^‡^	P value^§^		Mean±SD^‡^	P value^§^
A	Pre	1	161.0±18.1	<0.001		96.1±7.1	<0.001
		2	149.4±20.0			92.6±10.5	
		3	142.7±20.1			87.0±11.6	
		4	142.4±16.8			90.0±8.9	
		5	137.4±15.1			83.4±9.9	
		6	133.0±14.0			83.3±6.6	
	Post	1	146.0±17.7	<0.001		89.1±9.5	<0.001
		2	140.2±17.9			87.2±10.3	
		3	137.1±16.9			86.1±9.6	
		4	133.1±15.5			84.0±7.9	
		5	128.0±12.3			81.2±9.1	
		6	128.5±13.5			80.2±7.4	
B	Pre	1	160.5±16.7	0.056		98.6±8.4	0.062
		2	148.3±22.6			91.7±10.5	
		3	149.9±19.2			92.0±10.5	
		4	150.1±17.2			91.7±8.6	
		5	151.9±18.1			92.4±9.8	
		6	154.8±22.5			94.7±7.1	
	Post	1	154.2±17.1	0.59		95.4±8.8	0.179
		2	153.2±17.4			92.7±9.6	
		3	152.6±18.5			92.5±8.5	
		4	152.1±16.6			92.6±8.3	
		5	149.9±21.4			91.7±12.2	
		6	156.9±20.7			95.7±7.3	

*SBP: Systolic blood pressure; †DBP: Diastolic Blood Pressure; ‡SD: Standard deviation; §Obtained by Anova for repeated measures

By comparing the moments between 1 and 6, it was observed a significantly higher variation in BP reduction in arm A than that observed in arm B. These results are shown in Table 3.

**Table 3:** Comparison between the results obtained for SBP and DBP in both arms of the study. Rio de Janeiro, RJ, Brazil, 2015

Arm	Exposure	Moment	SBP*	P value^§^	DBP^†^	P value^§^
			Mean±SD^‡^		Mean±SD^‡^	
A	Pre	1	161.0±18.1	<0.001	96.1±7.1	<0.001
		6	133.0±14.0		83.3±6.6	
	Post	1	146.0±17.7	<0.001	89.1±9.5	<0.001
		6	128.5±13.5		80.2±7.4	
B	Pre	1	160.5±16.7	0.226	98.6±8.4	0.037
		6	154.8±22.5		94.7±7.1	
	Post	1	154.2±17.1	0.548	95.4±8.8	0.875
		6	156.9±20.7		95.7±7.3	

*SBP: Systolic blood pressure; †DBP: Diastolic Blood Pressure; ‡SD: Standard deviation; §Obtained by Student’s t-test

No undesirable effects or adverse events that required the need to exchange participants between groups or compromised the quality of the study were identified or reported.

## Discussion

This study confirmed the hypothesis that AP, combined with drug treatment, has efficacy in the acute control of BP in patients with difficulty controlling their blood pressure levels. In the intervention arm, blood pressure dropped to levels below 140x90 mmHg, which may contribute significantly to the non-aggravation of the disease, as demonstrated in studies using other therapies for BP control^7^ ^,^ ^11^ ^-^ ^13^.

The technique proposed in this study, combined with drug treatment strategies, complemented the conventional treatment, confirming the potential of AP as an integrative and complementary health therapy, not as a substitute or alternative method to the current treatment models.

It is considered that nurses specialized in AP, by incorporating it as a nursing care technology, may help hypertensive patients to effectively control their BP, since its use helps to achieve the therapeutic goal for BP. This favors the minimization of risks and comorbidities, since the effective control of BP levels helps reducing cardiovascular risk, especially in non-diabetic hypertensive patients without renal disease^13^ ^-^ ^15^.

The observed decrease in both SBP and DBP was significant from a statistical point of view. Studies using other protocols and traditional AP techniques have shown better results in the reduction of SBP, although DBP was also benefited^7^ ^,^ ^15^ ^-^ ^16^.

Results showing an improvement in BP values, but with a very small reduction in its levels, as observed in Arm B (Table 2), were also noticed in control groups of other studies in which AP was used^14^ ^-^ ^15^. It is believed that this may be related to the posture adopted by the participants during the course of the research, by assuming a commitment with the treatment, especially those with failures in the drug treatment due to low adherence or forgetfulness. The care component, provided in the relationship between the health and research team and the patient, may also have had an impact. 

It seems reasonable to consider that the strengthening of the patient/health professionals bond is favored because of the need for weekly attendance at the health unit to perform the procedure. In addition, there has been a significant increase in BP monitoring, making it possible the early recognition of the need for adjustments in the medication doses and other non-drug interventions. This favors health promotion and prevention of diseases on which nurses have a significant contribution and participation, as observed in the conversations on arterial hypertension that occurred throughout the study. 

It should be noted that longitudinal effects, after discontinuation of therapy, have not yet been addressed, which is a limiting factor of this research for generalizing the results, a similar situation also found in other studies on the efficacy of the method^14^ ^-^ ^15^.

However, the results of this study indicate that people of both sexes, in an age group with predominance of SAH, without complications, undergoing the recommended drug treatment, who do not participate in special programs of physical activity or diets and with difficulty in controlling their blood pressure levels, may benefit from this type of intervention. It is worth stressing that this clientele profile is very common, especially in the area of basic health care.

Another relevant aspect is the use of protocols in health practices aimed at a single and holistic care, as is the case of AP, inserted in the list of Integrative and Complementary Health Practices (CIP). It is noteworthy that the adoption of predefined terapy protocols with the use of AP in its different techniques is an important limitation to be overcome, especially in clinical studies, given the need to prove the efficacy of these techniques. 

In this context, in order to overcome the aforementioned limitation and, at the same time, contemplate the fundamental characteristics of the RTC method and the theoretical-philosophical principles of the TCM/AP, the protocol elaborated in this study was developed based on the human processes frequently affected in people with SAH, according to the different interpretations of SAH by the TCM. Therefore, it is inferred that the reasoning used for the development of this protocol has positively favored the achievement of the results.

When participants with physical activity habits and on programmed diets for weight loss were considered as exclusion criteria, a possible bias in the reduction of BP produced by physical activity habits and weight loss was avoided. Similar strategies were observed in clinical trials analyzed in systematic review studies that investigated the use of AP for the treatment of SAH^6^ ^,^ ^15^.

Participants’ body weight and body mass index were investigated at initial and final nursing consultations and no significant changes were observed that could contribute to BP alterations. The same reasoning was used for the exclusion of smokers and alcoholics, considering that possible interruption of smoking and alcohol consumption could interfere in the reduction of blood pressure levels.

The exclusion of the use of other CIP by participants during the intervention phase was also considered in order to avoid possible bias, as the use of other CIP, concomitant with drug treatment, may also influence the BP levels, just like LA^17^ ^-^ ^18^. This was not observed in the participants of both arms who used the proposed therapy as the only CIP.

The limitations of this study include the difficulty in controlling participants’ lifestyles, the sample losses that occurred throughout the study related to personal issues of participants, the non-measurement of responses to therapy in a longer time interval and/or less regularity in the performance of the interventions, and the effectiveness of the results achieved after therapy discontinuation. However, these limitations did not compromise the quality of the study and the obtained results.

Therefore, it is considered that the results observed here contribute to the improvement of scientific knowledge in Nursing since they show a new therapeutic possibility for intervention and care of people with arterial hypertension, to be performed by acupuncturist nurses or recommended by non-acupuncturist nurses who recognize this practice as a possible method for the care of their patients. 

## Conclusion

Based on the results, it was possible to confirm the efficacy of the protocol. There was an acute reduction and a significant BP control in all participants of arm A during the intervention period, indicating the possibility of using this technology in the care of hypertensive patients. In order to further deepen and better understand the results of the protocol in a long term, as well as the improvement of BP levels after its discontinuation and other clinical benefits, it is recommended that further studies be conducted.
